# Risk factors for macular pucker after rhegmatogenous retinal detachment surgery

**DOI:** 10.1038/s41598-021-97738-x

**Published:** 2021-09-14

**Authors:** Toshiaki Hirakata, Yoshimune Hiratsuka, Shutaro Yamamoto, Koki Kanbayashi, Hiroaki Kobayashi, Akira Murakami

**Affiliations:** 1 Department of Ophthalmology, Juntendo University Faculty of Medicine, Hongo 3-1-3, Bunkyo-ku, Tokyo, 113-8421 Japan; 2 Hatori Eye Clinic, Gunma, Japan

**Keywords:** Medical research, Risk factors

## Abstract

Macular pucker, also known as an epiretinal membrane, sometimes forms after surgical repair of a rhegmatogenous retinal detachment (RRD) and can decrease visual acuity and cause aniseikonia. However, few reports are evaluating the risk factors of macular pucker using multivariate analysis. To evaluate the risk factors for macular pucker after RRD surgery, 226 patients who underwent RRD surgery and were monitored for greater than 12 months (23.2 ± 6.4 months) after surgery were analyzed retrospectively. Of these cases, macular pucker developed in 26 cases. Multiple logistic regression models of 22 clinical characteristics were performed. An increased risk of macular pucker after RRD surgery was significantly associated with preoperative vitreous haemorrhage (Odds ratio (OR), 4.71; 95% CI 1.19–18.62), multiple retinal breaks (OR, 8.07; 95% CI 2.35–27.71), re-detachment (OR, 19.66; 95% CI 4.87–79.38), and retinal detachment area (OR, 12.91; 95% CI 2.34–71.19). Macular pucker was not associated with the surgical technique. Regardless of the surgical technique used, careful observation for postoperative macular pucker is needed after RRD surgery in high-risk cases. These findings can be used to improve the surgical management of patients with RRD. (183 words).

## Introduction

Epiretinal membrane (ERM) can form due to multiple aetiologies. ERM can be idiopathic or occur after a retinal break, a rhegmatogenous retinal detachment (RRD) surgery, retinal inflammation, or photocoagulation. ERM formation after retinal reattachment surgery is referred to as macular pucker^1^. Macular pucker occurs secondary to an overlying preretinal membrane, which wrinkles the neurosensory retinal surface, leading to visual acuity loss, metamorphopsia, and aniseikonia^1^. Often, macular pucker necessitates an additional surgery to remove the membrane and correct these visual deficits.

Therefore, elucidating the risk factors for macular pucker is important in the post-surgical management and outcomes of patients with RRD. Several prior studies have assessed risk factors for macular pucker after RRD surgery^1–5^. However, many studies have only examined whether or not there is an association between macular pucker formation and back ground clinical characteristics, and have not assessed the strength of the association (e.g., relative risk or odds ratio). Moreover, the development of macular pucker and background clinical characteristics are interrelated, and one-to-one univariate analyses do not allow for an independent assessment of the association between background clinical characteristics and macular packer formation. Although a previous prospective study reported the risk factor of macular pucker by multivariate analysis, some characteristics have not been assessed as potentially correlated with outcomes^6^, such as past history of laser photocoagulation, preoperative lens status, use of a scleral buckle, use of silicone oil tamponade, and intraoperative complications.

Additionally, there are two surgical techniques, pars plana vitrectomy (PPV) and scleral buckle, that can be used either together or separately to repair RRD; however, to the best of our knowledge, no study has investigated the risk factors of postoperative macular pucker after RRD surgery as a whole (PPV and scleral buckle). Therefore, in the present study, we aimed to analyse the risk factors for macular pucker after RRD surgery using multivariate analyses by evaluating 22 potential clinical characteristics and surgical methodology.

## Results

A total of 226 patients with RRD (226 eyes) who underwent RRD surgery at Juntendo University from February 2013 to April 2015 and were followed-up for more than 12 months (23.2 ± 6.4 months) were included in this study. Patient characteristics are shown in Table 1. The patients comprised 149 male (65.9%) and 77 female patients (34.1%), averaging 51.3 ± 15.9 years of age. Of these 226 cases, macular pucker developed in 26 cases (11.5%; 95% confidence interval [CI] 7.65–16.40) during follow-up. The time between symptom onset and RRD surgery was 14.4 ± 15.8 days in the macular pucker group and 7.7 ± 7.4 days in the non-macular pucker group. The surgical technique used was as follows: PPV, 88 (35.8%); SBs, 89 (39.4%); and EBs, 49 (21.7%). PPV included PPV only, 81; and PPV + EBs, 7.

**Table 1:** Demographic and baseline clinical characteristics of patients undergoing RRD surgery.

	Macular pucker	Non-macular pucker	Total
Number	26	200	226
Age (SD)	55.1 ± 12.6	50.8 ± 16.2	51.3 ± 15.9
Sex, male (%)/female (%)	20 (76.9)/6 (23.1)	129 (64.5)/71 (35.5)	149 (65.9)/77 (34.1)
Time lag (day) (SD)	14.4 ± 15.8	7.7 ± 7.4	8.4 ± 9.0
Axial length, mm (SD)	26.1 ± 2.5	26.0 ± 2.2	26.0 ± 2.2
Past history of laser photocoagulation (%)	2 (7.7)	4 (2.0)	6 (2.7)
Phakic (%)/Psueudophakic (%)	23 (88.5)/3 (11.5)	173 (86.5)/27 (13.5)	196 (86.7)/30 (13.3)
Re-detachment (%)	10 (38.5)	6 (3.0)	16 (7.1)
Preoperative vitreous haemorrhage (%)	7 (26.9)	17 (8.5)	24 (10.6)
Preoperative macular detachment (%)	12 (46.2)	55 (27.5)	67 (29.6)
**Type of retinal break**			
Hole (%)	3 (11.5)	41 (20.5)	44 (19.5)
Retinal tear (%)	22 (84.6)	154 (77.0)	176 (77.9)
Large break (%)	1 (3.9)	5 (2.5)	6 (2.7)
Multiple retinal breaks (≥ 3) (%)	10 (38.5)	19 (9.5)	29 (12.8)
Position of major break (inferior/superior)	3 (11.5)/23 (88.5)	31 (15.5)/169 (84.5)	34 (15.0)/192 (85.0)
Retinal detachment area (≥ 3 quadrants) (%)	7 (26.9)	5 (2.5)	12 (5.3)
**Operation method**			
PPV (%)	13 (50.0)	75 (37.5)	88 (38.9)
SB (%)	7 (26.9)	82 (41.0)	89 (39.4)
EB (%)	6 (23.1)	43 (21.5)	49 (21.7)
Intraoperative cataract surgery (%)	10 (38.5)	47 (23.5)	57 (25.2)
Cryotherapy (%)	18 (69.2)	145 (72.5)	163 (72.1)
Gas tamponade (%)	21 (80.8)	137 (68.5)	158 (69.9)
Silicone oil tamponade (%)	5 (19.2)	14 (7.0)	19 (8.4)
Intra-operative complications (%)	1 (3.9)	12 (6.0)	13 (5.8)

EB, encircling buckle; IOL, intra ocular lens; PPV, pars plana vitrectomy; RRD, rhegmatogenous retinal detachment; SB, segmental buckle.

By univariate analysis, five factors, including retinal re-detachment (OR 20.21; 95% CI 6.51–62.76; p < 0.01), preoperative VH (OR 4.01; 95% CI 1.49–10.84; p = 0.01), multiple retinal breaks (OR 5.95; 95% CI 2.37–14.95; p < 0.01), RRD area spanning more than three quadrants (OR 14.37; 95% CI 4.16–49.68; p < 0.01), and SO tamponade (OR 3.16; 95% CI, 1.04–9.66; p = 0.04) were significantly associated with the development of macular pucker (Table 2). Furthermore, prior history of laser photocoagulation (OR 4.08; 95% CI 0.71–23.49; p = 0.12) and preoperative macular detachment (OR 2.26; 95% CI 0.98–5.19; p = 0.06) were associated with the occurrence of macular pucker but did not reach statistical significance in our patient cohort. By contrast, age, sex, axial length, lens status, type of break, causative break position, surgical technique, cryotherapy, gas tamponade, and intraoperative complications were not associated with the development of macular pucker.

**Table 2:** Univariate analysis of risk factors for macular pucker.

	Odds ratio	(95% CI)	p-value
Age	1.02	(0.99–1.05)	0.20
Sex (male/female)	1.83	(0.70–4.78)	0.21
Axial length (mm)	1.02	(0.84–1.24)	0.86
Past history of laser photocoagulation	4.08	(0.71–23.49)	0.12
Lens status (pseudophakic/phakic)	0.84	(0.23–2.97)	0.78
Re-detachment	20.21	(6.51–62.76)	< 0.01
Preoperative vitreous haemorrhage	3.97	(1.46–10.77)	< 0.01
Preoperative macular detachment	2.26	(0.98–5.19)	0.06
**Type of retinal break**			
Tear/hole	1.95	(0.56–6.85)	0.30
Giant tear/hole	2.73	(0.24–31.55)	0.42
Multiple retinal breaks (≥ 3)	5.95	(2.37–14.95)	< 0.01
Position of a major break (inferior/superior)	0.71	(0.20–2.51)	0.60
Retinal detachment area (≥ 3 quadrants)	14.37	(4.16–49.68)	< 0.01
**Operation method**			
EB/PPV	0.49	(0.19–1.30)	0.15
SB/PPV	0.81	(0.29–2.27)	0.68
Intraoperative cataract surgery	2.03	(0.87–4.78)	0.10
Cryotherapy	0.85	(0.35–2.08)	0.73
Gas tamponade	1.93	(0.70–5.36)	0.21
Silicon oil tamponade	3.16	(1.04–9.66)	0.04
Intra-operative complications	0.63	(0.08–5.03)	0.66

EB, encircling buckle; PPV, pars plana vitrectomy; SB, segmental buckle.

In the multivariable logistic regression model, preoperative VH (OR 4.71; 95% CI 1.19–18.62; p = 0.03), re-detachment (odds ratio 19.66; 95% CI 4.87–79.38; p < 0.01), multiple retinal breaks (OR 8.07; 95% CI 2.35–27.71; p < 0.01), and retinal detachment area (odds ratio 12.91; 95% CI 2.34–71.19; p < 0.01) were significantly associated with postoperative macular pucker formation (Table 3).

**Table 3:** Multivariate analysis of risk factors for macular pucker.

	Odds ratio	(95% CI)	p-value
Preoperative vitreous haemorrhage	4.71	(1.19–18.62)	0.03
Re-detachment	19.66	(4.87–79.38)	< 0.01
Multiple retinal breaks (≥ 3)	8.07	(2.35–27.71)	< 0.01
Retinal detachment area (≥ 3 quadrants)	12.91	(2.34–71.19)	< 0.01

## Discussion

The present study determined the risk factors for macular pucker formation after RRD surgery with consideration of various clinical characteristics and patient demographics. In our study, macular pucker developed in 26 of the 226 RRD cases (11.5%). Multivariate analysis revealed that risk factors for the development of macular pucker after RRD surgery were preoperative VH, postoperative re-detachment, presence of multiple retinal breaks, and retinal detachment area. Interestingly, there was no significant difference in occurrence of macular pucker when comparing the surgical techniques (vitrectomy vs. scleral buckle). Our results suggest that, regardless of the surgical technique used to repair RRD, careful follow-up is necessary in patients with identified risk factors of postoperative macular pucker.

Postoperative re-detachment was the most significant risk factor for macular pucker (OR: 19.66). This is consistent with prior findings from Lobes & Burton, which demonstrated that the need for multiple reattachment surgeries was associated with macular pucker^2^. Additionally, the present study identified that multiple retinal breaks were associated with an increased risk of macular pucker (OR: 8.07). This is supported by the findings of Heo et al. that also reported an increased risk of macular pucker with multiple retinal breaks^1^. Conversely, there were no significant associations between macular pucker formation and the area of retinal detachment or the presence of preoperative macular detachment.

Similar to our study, preoperative VH was previously reported in both prospective and retrospective studies as a risk factor for macular pucker after RRD surgery^1,3,6^. Hagler & Aturaliya demonstrated that 15 of 44 eyes with macular pucker (34%) had VH^7^. Shultz et al. reported that ERM formation occurred in 14 of 18 eyes in Terson syndrome patients who were followed up for 3 or more years^8^. Although PPV eliminates the VH during surgery, there were no significant differences in the incidence of macular pucker between PPV and scleral buckle surgery in the present study. Indeed, one prospective report previously examined risk factors of ERM after 25-gauge PPV by multivariate analyses and reported that VH was a significant risk factor of postoperative ERM at the one year follow-up^6^. Moreover, the rate of macular pucker after RRD surgery (12.1%, including only PPV) in the aforementioned study^6^ was slightly higher than that in the present study (11.5%; including PPV and scleral buckle). These findings indicate that the presence of a preoperative VH is a strong risk factor for macular pucker.

Additionally, the risk of macular pucker increased with retinal detachment area (≥ 3 quadrants). Wider area of retinal detachment suggests an increased interval between detachment and reattachment surgery. Indeed, although the time interval between onset and surgery was excluded in the analysis in the current study, the time lag was longer in macular pucker group than in non-macular pucker group (Table 1). ERM contains a variety of cell types, including glial cells (Müller cells, astrocytes and microglia), hyalocytes, macrophages, retinal pigment epithelial (RPE) cells, fibroblasts and myofibroblasts^9,10^. Ogata et al. demonstrated that the presence of pigment epithelium in the vitreous was significantly higher in RRD cases than in cases of diabetic retinopathy or idiopathic macular holes^11^. Kampic et al*.* reported that RPE cells are more common in ERMs that occur secondary to a retinal break and/or RRD^12^. Macular pucker could form from RPE cells released through the retinal break into the vitreous cavity^13^. These findings suggest that wider retinal detachment area, suggesting longer time between surgery and onset, could increase the exposure of various cells, such as RPE cells and glial cells from a retinal break, to the surface of the retina. Because fibroblasts and macrophages originate from blood, VH allows a similar entry of these cell types into the vitreous. Interestingly, the difference in surgical techniques was not related to the risk of macular pucker in the present study. This supports the notion that macular pucker formation instead depends on pre-surgical factors, such as the release of various cells into the vitreous and the exposure time of these cells to the retinal surface.

Previous reports have demonstrated that ILM peeling during initial PPV surgery prevents postoperative macular puckers; however, the use of ILM peeling is not standard in PPV surgery due to conflicting results in literature^6,14,15^. Park et al. reported that PPV with ILM peeling prevents postoperative macular pucker, and that ILM peeling does not cause deleterious effects^15^. On the other hand, Lim et al. showed decreased P1 amplitudes in multifocal electroretinograms in patients that received ILM peeling^16^. Additionally, after a 12-month follow-up, the mean retinal sensitivity in the 4° central area showed a greater and faster recovery in the ILM non-peeling group than in the ILM peeling group^17^. These findings underscore the importance of evaluating the risk factors for macular pucker to identify the cases that could benefit from ILM peeling during the initial RRD surgery, as certain risks do exist with this additional procedure. This study suggests that retinal surgeons should determine whether to use PPV with or without ILM peeling for patients with RRD based on risk factor assessment for macular pucker.

The advantages of our study included its relatively large sample size, long follow-up period, and the evaluation of multiple potential clinical characteristics (twenty-four clinical characteristics). Despite the strengths of the study, its limitations should be acknowledged as well. Firstly, due to the observational nature of the study, residual or unmeasured confounding factors could be possible; for example, other factors such as past history of uveitis, hydration during scleral buckling surgery, number of intraoperative photocoagulations, use of perfluorocarbon, and choroidal detachment were not evaluated. These factors could also affect the development of macular pucker after RRD surgery. Secondly, the inclusion criteria in this study included a follow-up period greater than 1 year; however, the length of follow-up time varied between cases (range, 366–1171 days). Future prospective studies with a longer and more uniform follow-up period are needed to further confirm the findings of the present study.

In summary, the present study investigated risk factors for macular pucker formation after RRD surgery by multivariate analysis. Preoperative VH, postoperative re-detachment, multiple retinal breaks, and wider retinal detachment area were associated with an increased risk of macular pucker formation after RRD surgery. There was no difference in macular pucker formation between patients who received PPV or scleral buckle. In patients with RRD having risk factors for macular pucker, performing surgery as soon as possible from onset of symptoms and including intraoperative ILM peeling may be recommended to prevent postoperative macular pucker formation. The findings of our study could be useful in improving the surgical management and outcomes of patients with RRD.

## Methods

Records for 226 consecutive eyes (226 patients) who were treated with PPV and/or scleral buckling for RRD repair between February 2013 and April 2015 were retrospectively reviewed. Only patients with a follow-up time of 12 months or longer were included in this review. This retrospective study was conducted in accordance with the Declaration of Helsinki and was approved by the Institutional Review Board of Juntendo University (16-292). All patients signed informed consent forms prior to the RRD surgery.

RRD surgery was performed using PPV, encircling buckle (EB), segmental buckle (SB), or PPV with EB. Internal limiting membrane (ILM) peeling was not performed with the initial surgery in any of the cases. Macular pucker was diagnosed as wrinkling of the inner retinal surface on ophthalmoscopy and spectral domain optical coherence tomography (SD-OCT; Carl Zeiss, Dublin, CA, and Heidelberg Engineering, Dossenheim, Germany). As in previous studies^6,18^, in the current study, macular pucker (postoperative ERM) was defined as hyperreflective lines above the inner retinal surface accompanied by deformation of the foveal pit of the OCT image.

Twenty-two clinical characteristics of patients with RRD were analysed, including the following: age (years), sex (yes/no), axial length (mm), past history of laser photocoagulation (yes/no), lens status (phakic or pseudophakic), incidence of re-detachment (yes/no), preoperative vitreous haemorrhage (VH) (yes/no), preoperative macular detachment (yes/no), type of retinal break (atrophic hole, retinal tear or large breaks), multiple retinal breaks (≥ 3 or < 3), break position (superior or inferior), RD area (≥ 3 quadrants or < 3 quadrants), surgery type (PPV, EB, or SB), cataract surgery with initial RRD operation (yes/no), cryotherapy (yes/no), gas tamponade (yes/no), silicone oil (SO) tamponade (yes/no), and intraoperative complications (yes/no). Major break position was classified as either superior or inferior to the horizontal meridian of the eye. The type of retinal break was defined as the most causative retinal break for RRD. Axial length was measured in 200 patients; it was not measured in 26 patients due to preoperative macular detachment or failure to perform the measurement.

Multiple logistic regression models were used to identify clinical characteristics associated with the incidence of macular pucker. First, each variable was analysed using a univariate model. Then, in order to measure the strength of association more reliably, we narrowed down the associated variables to be included in the multivariate analysis. In other words, only variables with a significance level (p < 0.01) in univariate analysis were included in a multivariate model. The threshold for statistical significance was p < 0.05 (two-tailed test). All statistical analyses were performed using Stata version 15 (StataCorp, College Station, TX, USA).

### Ethics approval

This retrospective study was approved by the Institutional Review Board of Juntendo University (16-292).
